# Plant Growth Stage Drives the Temporal and Spatial Dynamics of the Bacterial Microbiome in the Rhizosphere of *Vigna subterranea*

**DOI:** 10.3389/fmicb.2022.825377

**Published:** 2022-02-17

**Authors:** Caroline Fadeke Ajilogba, Oluwaseyi Samuel Olanrewaju, Olubukola Oluranti Babalola

**Affiliations:** ^1^Food Security and Safety Focus Area, Faculty of Natural and Agricultural Sciences, North-West University, Mmabatho, South Africa; ^2^Agricultural Research Council, Natural Resources and Engineering, Division of Agrometeorology, Pretoria, South Africa

**Keywords:** Bambara groundnut, microbiome function, pattern analysis, PGPR biomarkers, plant microbe interaction, underutilized legume, plant-growth dynamics, functional microbiome

## Abstract

Bambara groundnut (BGN) is an underutilized legume commonly found in sub-Saharan Africa. It thrives in marginal soils and is resistant to drought stress. Several studies have been carried out on the nutritional properties of BGN, but very little is known about the effects of plant growth changes and development on rhizosphere bacterial dynamics and function. This study reports on the bacterial dynamics and function in the bulk and rhizosphere soils of BGN at different growth stages (vegetative, flowering, pod-filling, and maturation stages). Aside from the maturation stage that shows distinct community structure from the other growth stages, results obtained showed no significant differences in bacterial community structure among the other growth stages. At a closer level, *Actinobacteria*, *Proteobacteria*, and *Acidobacteria* were dominant in rhizosphere soils at all growth stages. The bulk soil had the least average phyla abundance, while the maturity stage was characterized by the highest average phyla abundance. *Rubrobacter, Acidobacterium*, and *Skermanella* were the most predominant genus. It was observed from the analysis of operational taxonomic units that there was significant change in the bacterial structure of the rhizosphere with a higher abundance of potential plant growth-promoting rhizobacteria, at the different growth stages, which include the genera *Bacillus* and *Acidobacterium*. Biomarker analysis revealed 7 and 4 highly significant bacterial biomarkers by linear discriminant analysis effect size and random forest analysis at the maturation stage, respectively. The results obtained in this study demonstrated that the bacterial communities of BGN rhizosphere microbiome dynamics and function are influenced by the plant’s growth stages.

## Introduction

Rhizosphere bacterial communities are different across plant genotypes, locations, plant compartments, and plant growth stages (Edwards et al., 2015; Stopnisek and Shade, 2021). Bambara groundnut (BGN) [*Vigna subterranea* (L.) Verdc.] is a drought-tolerant, underutilized legume notable for its ability to thrive in marginal soils (Mayes et al., 2019; Olanrewaju et al., 2021b,c). Although studies have reported bacterial communities from various genotypes, compartments, and growth stages on many plants (Hamonts et al., 2018; Brown et al., 2020; Wang et al., 2020; Cordovez et al., 2021; Zheng et al., 2021), no such study is available on BGN.

The rhizosphere is a beehive of activities and interactions between plants, soil, and microorganisms in the soil (Nihorimbere et al., 2011). The plants, *via* their roots, release a wide range of chemical compounds and root exudates into the environment, which are used to attract beneficial microbes. These root exudates mediate the interactions between plants, microorganisms, and the environment (Badri et al., 2013; Chaparro et al., 2013). In the same way that root exudates affect the microbial community in the rhizosphere, as a result of the category of compounds secreted, microbes also have a way of determining the compounds in the rhizosphere by the type of volatiles or metabolites they secrete into the plant–root environment (Huang et al., 2014). This means that the activities of plant roots alter the biochemical environment of the soil, which in turn affects the categories of microbes in the soil at different seasons in the growth stages of plants (Shi et al., 2015). High-throughput sequencing (e.g., Illumina and 454 pyrosequencing) of 16S rRNA gene amplicons has been observed to enhance exploratory analysis of the structure of microbial communities, their taxon compositions, and phylogeny. The description of the root-associated microbial community diversity is better enhanced using high-throughput sequencing (Caporaso et al., 2011; Berendsen et al., 2012; Fierer et al., 2012; Peiffer et al., 2013; Philippot et al., 2013).

It was hypothesized that plant growth stages alter rhizosphere microbial dynamics and select for specific microbes through the release of exudates and rhizodeposits (Chaparro et al., 2012; Huang et al., 2014). This selection can be in response to pathogen attack (Berendsen et al., 2012), abiotic stress (Liu et al., 2020), low available nutrients (Guyonnet et al., 2018), and general plant health (Olanrewaju et al., 2019). In this study, we tested this hypothesis by analyzing the rhizosphere microbial composition of BGN at four distinct physiological growth stages; vegetative, flowering, pod-filling, and maturation. To the best of our knowledge, no metagenomic studies to determine the bacterial dynamics of BGN as affected by the growth stages have been reported. Being an underutilized legume, it increases the importance of this study to enable improved production of this crop for increased food security. Furthermore, microbiome function was predicted from the bacterial diversity present at each growth stage.

## Materials and Methods

### Bulk Soil and Rhizosphere Soil Samples Collection

Samples were collected in the bulk soil (designated as 0) in October, just before planting, and during plant developmental stages in November (N), December (D), January (J), and February (F) of 2015/2016 from the North-West University agricultural farm, Mafikeng campus (Lat. 25°82′18′′, Long. 2561′44′′) Mafikeng, South Africa, corresponding to BGN vegetative (4WAP), flowering (8WAP), pod-filling (12WAP), and maturation stage (16WAP), respectively. For each time point of the developmental/growth stage, rhizosphere soil was collected from each plot following a destructive sampling method in which the plants were uprooted and the soil samples attached to the roots were gently shaken off into a plastic bag. In total, 20 samples [2 samples from the bulk soil designated as 01 and 04, 2 samples each from the rhizosphere soils at the vegetative growth stage (N1 and N4), flowering stage (D1 and D4), pod-filling stage (J1 and J4), and maturation stage (F1 and F4)] were collected. Two collections were made for bulk soil and the rhizosphere stages. The bulk soil sample was collected at the beginning of the experiment (1) and after 4 weeks (4), while the rhizosphere soils were collected at the beginning of each stage and toward the end, designated as 1 and 4, respectively. Collected samples were transported to the lab on dry ice and stored at −80°C until further processing and analysis.

### DNA Extraction and PCR Amplification

The MOBIO PowerSoil^®^ DNA Isolation Kit was used to extract DNA as directed (MO BIO Laboratories, Inc., Carlsbad, CA, United States), and all DNA samples were frozen until further analysis by the Molecular Research DNA analysis (MR DNA, Shallowater, TX, United States). To target the 16S rRNA gene for region V3 and V4, PCR primers 515/806 (Caporaso et al., 2011) with barcoding on the forward primer were employed in a 28 cycle PCR (5 cycles on PCR products) using the HotStar Taq Plus Master Mix Kit (Qiagen, Germantown, TN, United States). The PCR amplification was performed at 94°C for 3 min, then 28 cycles of 94°C for 30 s, 53°C for 40 s, and 72°C for 1 min, followed by a final elongation step for 5 min at 72°C.

### Data Sequencing and Processing

We further checked PCR products in 2% agarose gel to determine the relative intensity of bands. The PCR products were pooled in equal proportions on their molecular weight and concentrations and Illumina DNA library was subsequently prepared using the calibrated Ampure beads-purified PCR products. Sequencing was performed at MR DNA (^1^ Shallowater, TX, United States) on a MiSeq following the manufacturer’s guidelines, and generated data were processed using their analysis pipeline.

### Bioinformatic Pipeline for Analysis of 16S Sequencing Dataset

Sequenced data were derived by sequencing the V3–V4 region of the 16S rRNA gene as described at MR DNA Laboratory (see text footnote 1). Barcodes, primers, sequences with ambiguous calls, and sequences less than 150 bp were removed. Subsequent removal of homopolymer runs (>6 bp) was followed by denoising, generation of operational taxonomic units (OTUs), and removal of chimeric sequences and all abnormal sequences were removed. A 3% divergence (97% similarity) was used to filter the OTUs at the species level and were further classified according to their taxonomy into ‘‘counts’’ and ‘‘percentage’’ files using BLASTn against a curated database derived from RDPII and NCBI^2^ ^,3^. Sequences >97% identity were counted for the species. At the genus, family, order, class, and phylum levels, consideration was based on the sequences having identities between 95 and 97%; 90 and 95%; 85 and 90%; 80 and 85%; and those between 77 and 80%, respectively (Mills et al., 2012).

### Statistical and Network Analysis

All analyses were conducted using the MicrobiomeAnalyst Platform^4^ (Dhariwal et al., 2017; Chong et al., 2020). Microbiome Analyst is a comprehensive statistical, visual, and meta-analysis tool for microbiome data that utilizes the Microbiome Analyst R package for statistical analysis and graphical outputs. From the data that were passed to the MicrobiomeAnalyst pipeline, low abundant features were filtered based on the mean values with the minimum count set at 4 while the low variance features were removed based on the interquartile range. After data filtering, the remaining features were normalized using the total sum scaling (TSS) method before further analysis. Alpha diversity was calculated using the chao1 and Shannon index. For the beta diversity and significant testing, the PCoA ordination method was used with the Bray–Curtis index at the species level with PERMANOVA applied as the statistical method. The core microbiome analysis was conducted at the genus level with a sample prevalence of 20 and a relative abundance of 0.01. Furthermore, we performed a pattern search to identify microbiome patterns at all growth stages using the SparCC correlation at the species level. The linear discriminant analysis (LDA) and random forest (RF) methods were used for the biomarker analysis. The platform performs non-parametric factorial Kruskal–Wallis sum–rank test to identify features with significant differential abundance considering the class of interest, followed by LDA to calculate the effect size of each differentially abundant features. The features are considered significant depending on their adjusted *p*-value. The default adj.*p*-value cutoff = 0.05 and the LDA score is 2.0. The randomForest package5 was further used to perform the RF analysis. RF is a supervised learning algorithm that is suitable for high-dimensional data analysis. This method uses an ensemble of classification trees, each of which is grown *via* random feature selection from a bootstrap sample at each branch (Dhariwal et al., 2017).

## Results

### Bacterial Microbiomes in the Bulk Soil and Rhizosphere Soils Across Plant Developmental Stages

**FIGURE 1:**
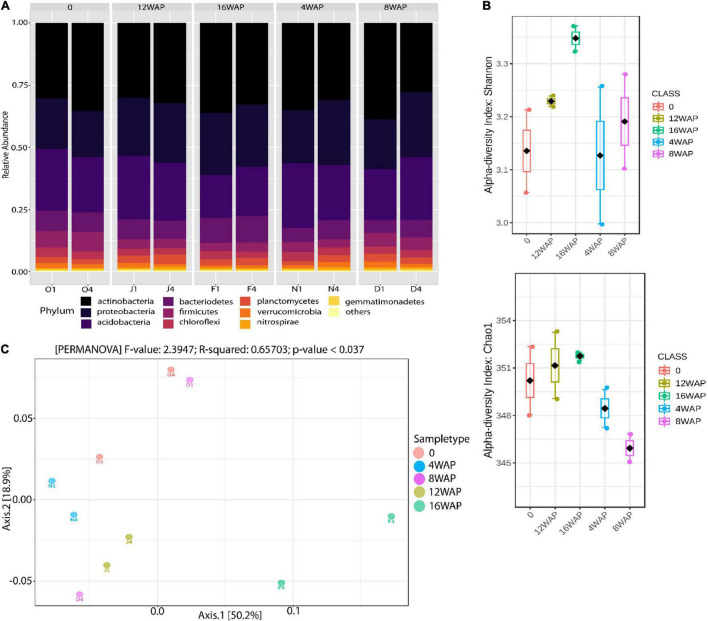
Rhizobacterial community assembly of Bambara groundnut plants during their growth cycle. **(A)** Taxonomic distribution of bacterial communities in the rhizosphere soils at different growth stages and in the bulk soil. **(B)** Alpha diversity of 16S rRNA rhizosphere bacterial sequences of Bambara groundnut in the rhizosphere soils at different growth stages. Shannon and Chao 1 diversity indices were calculated with Total Sum Scaling (TSS) normalized counts. **(C)** Principal Coordinate Analysis (PCoA) of 16S rRNA diversity in the rhizosphere of the Bambara groundnut growth stages and the bulk soil.

The relative abundance of bacterial phyla and species detected across all plant growth stages is shown in Figure 1A and Supplementary Tables 1, 2. Taxonomic classification showed that bacterial communities in the bulk and rhizosphere soils varied across the four developmental stages (Figure 1A). *Actinobacteria* (from 27.84 to 38.72%), *Proteobacteria* (from 18.67% to 26.17%), and *Acidobacteria* (from 17.3% to 26.05%) represented more than 60% of the total bacteria detected (Figure 1A and Supplementary Table 1). The most abundant families were *Rubrobacteriaceae* (from 19.84% to 30%), *Acidobacteriaceae* (from 15% to 25.33%), and *Rhodospirillaceae* (from 3.42% to 7.65%) (Supplementary Table 2). The ANOVA and *t*-test analysis suggested that plant developmental stage had a greater influence on bacterial Chao 1 and Shannon richness in the rhizosphere (especially at 16WAP, which corresponds to seed maturation stage) than the bulk soil (Figure 1B and Supplementary Figure 1). Both bacterial Chao 1 and Shannon richness were similar for the rhizosphere soils at flowering and pod filling stages but lower at the vegetative stage (Figure 1B). However, the highest values for both diversity indices were recorded in the rhizosphere soils sampled at the maturation stage of the plant. Remarkably, both ANOVA and *t*-test revealed no significant effect of plant growth stage and soil type on the diversity indices (*p* < 0.05) (Supplementary Table 3). PERMANOVA analysis indicated that plant growth stages were the primary source of beta diversity (*R*^2^ = 0.66, *p* < 0.037) (Figure 1C). Axis 1 is influenced by diversity in the rhizosphere soil samples at the maturity and flowering stages while axis 2 is influenced by observed diversities at the flowering and vegetative stages of the rhizosphere soil samples (Supplementary Table 4). On the other hand, the bulk soil had little influence on axis 1 and a more pronounced influence on axis 2 (Supplementary Table 4).

### Temporal Dynamics of Bacterial Rhizobiome Composition Across Soil Niches

**FIGURE 2:**
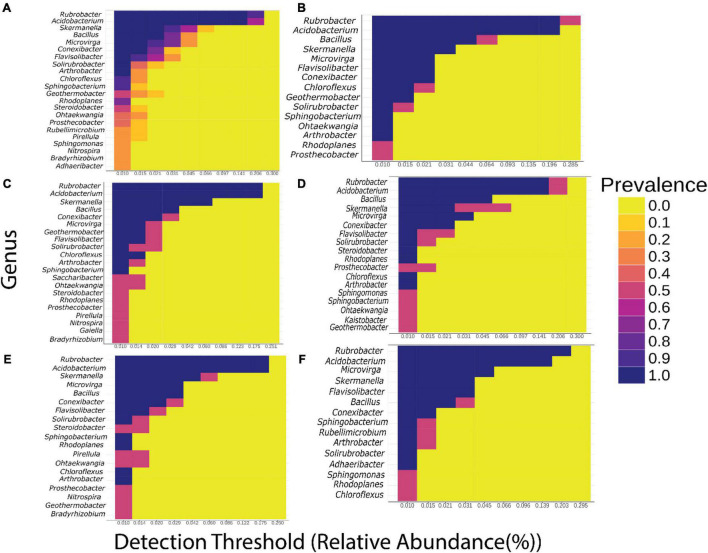
Heatmap of the core bacterial genera. *x*-axis, relative abundance; colors indicate the prevalence in the sample. **(A)** Rhizosphere soil, **(B)** bulk soil [0], **(C)** vegetative growth stage [4WAP], **(D)** flowering stage [8WAP], **(E)** pod-filling stage [12WAP], and **(F)** maturation stage [16WAP].

Nine bacteria genera were identified as core at a prevalence threshold of 100% across bulk and rhizosphere soils and across plant growth stages (Figure 2 and Supplementary Table 5). The BGN core microbiome primarily included *Rubrobacter*, *Acidobacterium*, *Skermanella*, *Bacillus*, *Microvirga*, *Conexibacter*, *Flavisolibacter*, *Solirubrobacter*, and *Arthrobacter* genera. In the rhizosphere soil, *Sphingobacterium* and *Chloroflexus* were found at 88% threshold prevalence, while *Rhodoplanes*, *Steroidobacter*, and *Geothermobacter* were present at 75, 50, and 50%, respectively (Figure 2A and Supplementary Table 5). Interestingly, in addition to the primary core microbiome genera, the bulk soil had *Ohtaekwangia* and *Prosthecobacter* at 100 and 50%, respectively (Figure 2B and Supplementary Table 5). Furthermore, core genera associated with each growth stage were generally part of the overall core taxa at the 100% prevalence threshold. At all growth stages, *Chloroflexus*, *Sphingobacterium*, and *Rhodoplanes* were present in the rhizosphere soils in all growth stages, while *Geothermobacter*, *Steroidobacter*, *Ohtaekwangia*, and *Prosthecobacter* joined the core taxa in the rhizosphere soils at vegetative, flowering, and pod-filling stages (Figures 2C–E). *Pirellula*, *Nitrospira*, and *Bradyrhizobium* were observed only at the vegetative and pod-filling stages (Figures 2C,E), and *Sphingomonas* was observed at flowering and maturation stages (Figures 2D,F). *Rubellimicrobium* and *Adhaeribacter* were only present during maturation (Figure 2F), while *Saccharibacter* and *Gaiella* were only present in rhizosphere soils at the vegetative stage (Figure 2C) and *Kaistobacter* was present only in the flowering stage (Figure 2D).

### Bacterial Community Biomarkers Associated With Bambara Groundnut Growth Stages

**FIGURE 3:**
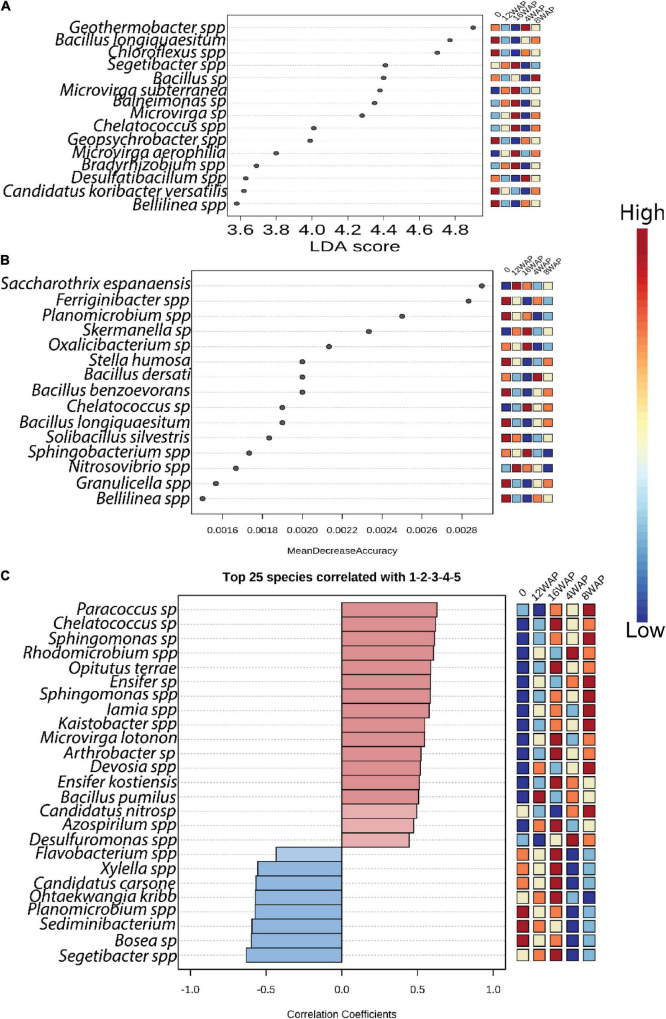
Bacterial biomarkers associated with bulk soil and plant growth stages. **(A)** LDA analysis indicating the differences in bacterial taxa among the growth stages **(B)** Random forest analysis indicating the differences in bacterial taxa among the growth stages. **(C)** Correlation pattern network analysis indicating the differences in bacterial taxa among the growth stages. Only predictors with significant effects are shown. Color from blue to red indicates from low to high level of significance.

Linear discriminant analysis (LDA) identified the major bacterial taxa that were significantly imparted by the growth stages (Figure 3A and Supplementary Table 6). Except for the pod-filling stage, some bacterial taxa were identified as significant markers in each stage. However, the bacterial communities tend to be less affected in the vegetative, flowering, and pod-filling stages with few bacterial taxa identified in these stages (Figure 3A). Interestingly, five bacterial markers were identified in the bulk soil. Two were identified in the vegetative stage, one was identified in the flowering stage, none was identified in the pod-filling stage, and seven were identified in the maturation stage (Figure 3A). Random forest analysis identified the flowering stage as having the least impact on the bacterial community because no taxa were identified as being significantly high in this stage (Figure 3B). Interestingly, eight bacterial taxa were significantly more abundant in the bulk soil when compared to the rhizosphere soils from the different growth stages, which together contained only seven bacterial taxa in high abundance (Figure 3B).

We further performed pattern correlation analysis to assess the dynamics of bacterial interactions across the plant growth stages (Figure 3C and Supplementary Table 7) along the plant–soil continuum. Only the top 25 significant correlations (*p* < 0.05) are shown. Our results showed that bacterial network patterns shifted clearly across the growth stages. Interestingly, the bulk soil had three significant negative correlations, the vegetative stage had two significant positive correlations, the flowering stage had eight significant positive correlations, the pod-filling stage had one significant positive correlation, and the maturation stage had eleven, with six being positive and five being negative correlations (Figure 3C and Supplementary Table 7).

### Patterns of Bacterial Diversity in the Bulk and Rhizosphere Soil Samples

**FIGURE 4:**
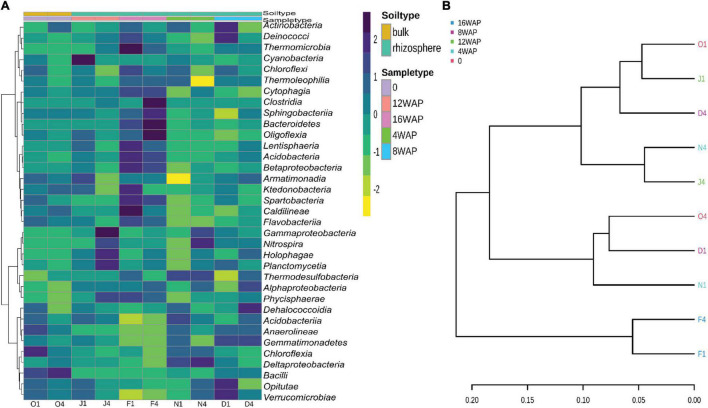
**(A)** Heatmap of the bacterial biota in the bulk and rhizosphere soil at the different developmental stages at the class level. **(B)** Phylogenetic tree showing the relationship between the bulk soil and rhizosphere soil samples at the class level.

The heatmap showed a high abundance of some taxonomy classes in the bulk soil, and in the rhizosphere soils at the different growth stages of the plant (Figure 4A). The purple color represents those taxonomic classes that were highly abundant while the yellow color represents the less abundant taxonomic classes in the rhizosphere soils at each growth stage. The abundance of *Chloroflexia* and *Bacilli* were abundant in the bulk soil, while the vegetative stage was characterized by a high abundance of *Nitrospira* and *Deltaproteobacteria* in the rhizosphere. Interestingly, the flowering stage was characterized by a high abundance of five taxonomic classes, while the pod filling and maturity stages were each characterized by a high abundance of four and eleven taxonomic classes, respectively (Figure 4A). Interestingly, the pattern analysis also identified eleven correlated patterns at the species level (Figure 3C). Clustering analysis of the bulk soil and the rhizospheric soils based on the identified taxonomy presented a result of mixed patterns in the clusters (Figure 4B). Only the maturation stage had a distinct cluster from the other stages. This supports the results from the heatmap analysis (Figure 4A), pattern network analysis (Figure 3C), LDA (Figure 3A), alpha (Figure 1A), and beta diversity analysis (Figure 1B), where it showed distinct attributes to other stages.

## Discussion

### Plant Developmental Stage Strongly Influences the Assembly of Plant Microbiomes

Defining the ecological principles and complex interactions in plant microbiome development is crucial to understanding the coevolution of plants with their biomes and the coevolution of inter-kingdom interactions in the plant microbiome for future application and introgression into plant breeding. Invariably, plants shape their microbiomes because of their responses to pests, low nutrients, drought, and salinity stress (Berendsen et al., 2012; Sharma and Verma, 2018; Korenblum et al., 2020). Plant developmental stages are also important in shifting plant microbiome diversity and function (Qiao et al., 2017). These changes are made possible through the release of root exudates, which serve as communication links between plants and their biomes (Berendsen et al., 2012; Olanrewaju, 2016; Olanrewaju et al., 2019). From our results, BGN microbiome assembly is highly influenced by the rhizosphere microbiome rather than the bulk soil microbiome. Furthermore, plant rhizobacterial communities respond differently to plant developmental stages than bulk soil bacterial communities based on multiple microbial attributes implemented in this study, such as alpha and beta diversities, community structure, biomarker analysis, and pattern network analysis. Our findings are consistent with previous results showing that plant developmental stages shape the assembly of plant microbiomes (Chaparro et al., 2014; Qiao et al., 2017; Cordovez et al., 2021; Ma et al., 2021; Xiong et al., 2021a).

This was similar to the report by Shi et al. (2015) who observed that the growth of plants drove the succession of rhizobacteria in the rhizosphere of *Avena fatua*. Li et al. (2014) observed changes in the maize rhizosphere bacterial communities between early and late growth stages, which were due in part to either the growth stages or seasonal effects. Chaparro et al. (2014) reported that rhizospheric bacterial communities of *Arabidopsis thaliana* at the seedling growth stage were distinct and different compared to the other growth stages. There was a difference between the bacterial community of the rhizosphere of soybean during growth, evident after 6 weeks corresponding to the vegetative stage compared to the bulk soil using pyrosequencing analysis at the phylum level (Sugiyama et al., 2014).

Furthermore, metagenomic analysis in our study revealed that the bacterial diversity in the BGN rhizosphere varied across the four developmental stages. The plant exerts a strong selection mechanism that recruits and selects for specific bacterial taxa during the plant’s development (Fazal et al., 2021; Sun et al., 2021). In addition to previous studies of plants shaping their microbiomes through the various compartments and varying genotypes (Agoussar et al., 2021; Stopnisek and Shade, 2021; Wagner, 2021; Xiong et al., 2021b), this study further identifies plant development stages as an important process in plant-engineered microbiome assembly and function.

The dynamics of plant developmental stages is a culmination of plant metabolism, plant metabolites released as exudates, volatiles, and other growth responses (Badri and Vivanco, 2009; Weits et al., 2021). These exudates, which differ in their chemical composition and function, act as signal molecules in recruiting microbes to the plant biome (Baetz and Martinoia, 2014; Olanrewaju et al., 2019; Rolfe et al., 2019). This study expanded our knowledge of the dynamics of the rhizosphere microbiome of BGN at various growth stages. To the best of our understanding, this is the first study of the rhizosphere microbiome dynamics of BGN in response to developmental stages. Being an underutilized legume, it further expands our knowledge for improved breeding through effective selection and application of beneficial plant growth-promoting bacteria to the crop.

### The Differentiation in Ecological Roles of Bacterial Communities Across Plant Developmental Stages

Bacteria and their hosts have coevolved for more than 400 million years (Martin et al., 2017); hence, they cannot be excluded from playing important roles in plant growth and development. In this study, bacterial interactions shifted across the four developmental stages. The bacterial community possessed higher alpha diversity and network correlations at the maturation stage. This implies that the plant host selectively modifies its bacterial interactions to meet the necessary requirements for nutrient and other growth-promoting needs at each growth stage. In plant development, the most crucial stage that affects yield is the flowering and pod-filling stage; hence, highly correlated bacteria taxa in this stage can be selected as effective biomarkers for improving crop yield in BGN (Figure 3). Several studies have suggested that rhizosphere bacteria can improve nutrient availability for plant growth (Babalola et al., 2019; Olanrewaju and Babalola, 2019a; Olanrewaju et al., 2021a). Due to the increase in diversity and correlation network pattern at the pod-filling and maturation stages, we can conclude that bacteria may play a more important ecological role at these stages than at the other growth stages. Our study supports the argument that plant hosts facilitate fitness and microbiome balance through precise selection during plant growth stages. These findings therefore provide new insights into plant–microbe interactions for application in rhizosphere microbiome engineering.

### Bacterial Taxa and Their Ecological Functions at Different Developmental Stages: Linking Bacterial Dynamics With Potential Function

The composition and potential functions of the microbiome change over plant growth stages, and more *Actinobacteria* and *Proteobacteria* were reported at the maturation stage than at the other stages of plant growth, while more abundant *Acidobacteria* were observed at the pod-filling stage than at the other growth stages. *Actinobacteria* are well known for their biocontrol abilities as a result of their high production of antibiotics against pathogens (Olanrewaju and Babalola, 2019a,b; Wonglom et al., 2019; Xu et al., 2019). Furthermore, rhizosphere soils were significantly enriched in the *Rubrobacteriaceae* family of the *Actinobacteria* class at all stages of development, but a bit higher at the maturation stage (Supplementary Table 7). *Rubrobacteriaceae* are highly desiccation-tolerant bacteria that have been shown to maintain a high intracellular concentration of the osmoprotectants trehalose and mannosylglycerate (Meier et al., 2021). In the absence of a sufficient amount of water, these osmolytes help in surviving desiccation. As a drought-tolerant plant, we propose high activity of the *Rubrobacteriaceae* family in the rhizosphere of BGN. This implies that the *Rubrobacteriaceae* are an active ally in preserving the plant during low water levels in the soil. The *Acidobacteriaceae* family, which were the second most abundant, are known for their ability to survive at low pH (Alves et al., 2018). Therefore, these families of bacteria are important in disease suppression. Our study demonstrates specificity in plant microbiome recruitment at different growth stages. Revealing the complex molecular mechanisms involved in this selection at various growth stages is important in deciphering the succinct roles of the microbiome in plant growth and adaptation to climate change impact and achieving sustainable agriculture. We also propose that further research involving root exudates at each growth stage should be examined to effectively link plant succinct roles of influencing microbiome dynamics at each growth stage.

## Conclusion

This study provides a systematic understanding of BGN microbiome composition and potential functions during plant development by examining the temporal dynamics of bacterial communities across soils and growth stages. Plant developmental stage influences multiple microbial attributes (alpha-diversity, community structure, markers, and correlation pattern networks) in the rhizosphere more than in the bulk soils. At the pod-filling and maturation stages, bacteria play a larger role in maintaining plant health through disease suppression and drought tolerance. From these findings, BGN appears to have a strong selective modulation effect on the composition and potential functions of plant microbiomes during their developmental stages. These findings provide critical new knowledge for future community research and the development of microbiome tools to enhance BGN production.

## Data Availability Statement

The datasets presented in this study can be found in online repositories. The names of the repository/repositories and accession number(s) can be found below: https://www.ncbi.nlm.nih.gov/, BioProject ID: PRJNA422360; BioSample accession: SAMN08176610.

## Author Contributions

All authors contributed equally to the manuscript.

## Conflict of Interest

The authors declare that the research was conducted in the absence of any commercial or financial relationships that could be construed as a potential conflict of interest.

## Publisher’s Note

All claims expressed in this article are solely those of the authors and do not necessarily represent those of their affiliated organizations, or those of the publisher, the editors and the reviewers. Any product that may be evaluated in this article, or claim that may be made by its manufacturer, is not guaranteed or endorsed by the publisher.

## Footnotes

1
www.mrdnalab.com

2
www.ncbi.nlm.nih.gov

3
http://rdp.cme.msu.edu

4
https://www.microbiomeanalyst.ca

## References

[B1] Agoussar A. Azarbad H. Tremblay J. Yergeau É (2021). The resistance of the wheat microbial community to water stress is more influenced by plant compartment than reduced water availability. *FEMS Microbiol. Ecol.* 97:fiab149. 10.1093/femsec/fiab149 34791186

[B2] Alves M. Pereira A. Vicente C. Matos P. Henriques J. Lopes H. (2018). The role of bacteria in pine wilt disease: insights from microbiome analysis. *FEMS Microbiol. Ecol.* 94:fiy077. 10.1093/femsec/fiy077 29718181

[B3] Babalola O. O. Ayangbenro A. S. Olanrewaju O. S. (2019). Draft genome sequences of three rhizospheric plant growth-promoting bacteria. *Microbiol. Resour. Announc.* 8 e455–e419. 10.1128/MRA.00455-19 31248993 PMC6597687

[B4] Badri D. V. Chaparro J. M. Zhang R. Shen Q. Vivanco J. M. (2013). Application of natural blends of phytochemicals derived from the root exudates of *Arabidopsis* to the soil reveal that phenolic-related compounds predominantly modulate the soil microbiome. *J. Biol. Chem.* 288 4502–4512. 10.1074/jbc.M112.433300 23293028 PMC3576057

[B5] Badri D. V. Vivanco J. M. (2009). Regulation and function of root exudates. *Plant Cell Environ.* 32 666–681. 10.1111/j.1365-3040.2008.01926.x 19143988

[B6] Baetz U. Martinoia E. (2014). Root exudates: the hidden part of plant defense. *Trends Plant Sci.* 19 90–98. 10.1016/j.tplants.2013.11.006 24332225

[B7] Berendsen R. L. Pieterse C. M. Bakker P. A. (2012). The rhizosphere microbiome and plant health. *Trends Plant Sci.* 17 478–486. 10.1016/j.tplants.2012.04.001 22564542

[B8] Brown S. P. Grillo M. A. Podowski J. C. Heath K. D. (2020). Soil origin and plant genotype structure distinct microbiome compartments in the model legume *Medicago truncatula*. *Microbiome* 8 1–17. 10.1186/s40168-020-00915-9 32988416 PMC7523075

[B9] Caporaso J. G. Lauber C. L. Walters W. A. Berg-Lyons D. Lozupone C. A. Turnbaugh P. J. (2011). Global patterns of 16s rRNA diversity at a depth of millions of sequences per sample. *Proc. Natl. Acad. Sci. U. S. A.* 108 4516–4522. 10.1073/pnas.1000080107 20534432 PMC3063599

[B10] Chaparro J. M. Badri D. V. Bakker M. G. Sugiyama A. Manter D. K. Vivanco J. M. (2013). Root exudation of phytochemicals in *Arabidopsis* follows specific patterns that are developmentally programmed and correlate with soil microbial functions. *PLoS One* 8:e55731. 10.1371/journal.pone.0055731 23383346 PMC3562227

[B11] Chaparro J. M. Badri D. V. Vivanco J. M. (2014). Rhizosphere microbiome assemblage is affected by plant development. *ISME J.* 8 790–803. 10.1038/ismej.2013.196 24196324 PMC3960538

[B12] Chaparro J. M. Sheflin A. M. Manter D. K. Vivanco J. M. (2012). Manipulating the soil microbiome to increase soil health and plant fertility. *Biol. Fertility Soils* 48 489–499. 10.1007/s00374-012-0691-4

[B13] Chong J. Liu P. Zhou G. Xia J. (2020). Using microbiomeanalyst for comprehensive statistical, functional, and meta-analysis of microbiome data. *Nat. Protoc.* 15 799–821. 10.1038/s41596-019-0264-1 31942082

[B14] Cordovez V. Rotoni C. Dini-Andreote F. Oyserman B. Carrión V. J. Raaijmakers J. M. (2021). Successive plant growth amplifies genotype-specific assembly of the tomato rhizosphere microbiome. *Sci. Total Environ.* 772:144825. 10.1016/j.scitotenv.2020.144825 33581524

[B15] Dhariwal A. Chong J. Habib S. King I. L. Agellon L. B. Xia J. (2017). Microbiomeanalyst: a web-based tool for comprehensive statistical, visual and meta-analysis of microbiome data. *Nucleic Acids Res.* 45 W180–W188. 10.1093/nar/gkx295 28449106 PMC5570177

[B16] Edwards J. Johnson C. Santos-Medellín C. Lurie E. Podishetty N. K. Bhatnagar S. (2015). Structure, variation, and assembly of the root-associated microbiomes of rice. *Proc. Natl. Acad. Sci. U. S. A.* 112 E911–E920. 10.1073/pnas.1414592112 25605935 PMC4345613

[B17] Fazal A. Yang M. Wen Z. Ali F. Ren R. Hao C. (2021). Differential microbial assemblages associated with shikonin-producing borage species in two distinct soil types. *Sci. Rep.* 11:10788. 10.1038/s41598-021-90251-1 34031500 PMC8144371

[B18] Fierer N. Leff J. W. Adams B. J. Nielsen U. N. Bates S. T. Lauber C. L. (2012). Cross-biome metagenomic analyses of soil microbial communities and their functional attributes. *Proc. Natl. Acad. Sci. U. S. A.* 109 21390–21395. 10.1073/pnas.1215210110 23236140 PMC3535587

[B19] Guyonnet J. P. Guillemet M. Dubost A. Simon L. Ortet P. Barakat M. (2018). Plant nutrient resource use strategies shape active rhizosphere microbiota through root exudation. *Front. Plant Sci.* 9:1662. 10.3389/fpls.2018.01662 30559748 PMC6265440

[B20] Hamonts K. Trivedi P. Garg A. Janitz C. Grinyer J. Holford P. (2018). Field study reveals core plant microbiota and relative importance of their drivers. *Environ. Microbiol.* 20 124–140. 10.1111/1462-2920.14031 29266641

[B21] Huang X.-F. Chaparro J. M. Reardon K. F. Zhang R. Shen Q. Vivanco J. M. (2014). Rhizosphere interactions: root exudates, microbes, and microbial communities 1. *Botany* 92 267–275.

[B22] Korenblum E. Dong Y. Szymanski J. Panda S. Jozwiak A. Massalha H. (2020). Rhizosphere microbiome mediates systemic root metabolite exudation by root-to-root signaling. *Proc. Natl. Acad. Sci. U. S. A.* 117 3874–3883. 10.1073/pnas.1912130117 32015118 PMC7035606

[B23] Li X. Rui J. Mao Y. Yannarell A. Mackie R. (2014). Dynamics of the bacterial community structure in the rhizosphere of a maize cultivar. *Soil Biol. Biochem.* 68 392–401. 10.1016/j.soilbio.2013.10.017

[B24] Liu H. Brettell L. E. Qiu Z. Singh B. K. (2020). Microbiome-mediated stress resistance in plants. *Trends Plant Sci.* 25 733–743. 10.1016/j.tplants.2020.03.014 32345569

[B25] Ma Z. Yi Z. Bayar K. Fu Y. Liu H. (2021). Community dynamics in rhizosphere microorganisms at different development stages of wheat growing in confined isolation environments. *Appl. Microbiol. Biotechnol.* 105 3843–3857. 10.1007/s00253-021-11283-1 33914137

[B26] Martin F. M. Uroz S. Barker D. G. (2017). Ancestral alliances: plant mutualistic symbioses with fungi and bacteria. *Science* 356:eaad4501. 10.1126/science.aad4501 28546156

[B27] Mayes S. Ho W. K. Chai H. H. Gao X. Kundy A. C. Mateva K. I. (2019). Bambara groundnut: an exemplar underutilised legume for resilience under climate change. *Planta* 250 803–820. 10.1007/s00425-019-03191-6 31267230

[B28] Meier D. V. Imminger S. Gillor O. Woebken D. (2021). Distribution of mixotrophy and desiccation survival mechanisms across microbial genomes in an arid biological soil crust community. *mSystems* 6:e00786-20. 10.1128/mSystems.00786-20 33436509 PMC7901476

[B29] Mills H. J. Reese B. K. Peter C. S. (2012). Characterization of microbial population shifts during sample storage. *Front. Microbiol.* 3:49. 10.3389/fmicb.2012.00049 22363327 PMC3281211

[B30] Nihorimbere V. Ongena M. Smargiassi M. Thonart P. (2011). Beneficial effect of the rhizosphere microbial community for plant growth and health. *Biotechnol. Agron. Soc. Environ.* 15 327–337.

[B31] Olanrewaju O. S. (2016). *Isolation of Bacterial Strains for Improved Maize Production.* Potchefstroom: North-West University.

[B32] Olanrewaju O. S. Ayangbenro A. S. Glick B. R. Babalola O. O. (2019). Plant health: feedback effect of root exudates-rhizobiome interactions. *Appl. Microbiol. Biotechnol.* 103 1155–1166. 10.1007/s00253-018-9556-6 30570692 PMC6394481

[B33] Olanrewaju O. S. Ayilara M. S. Ayangbenro A. S. Babalola O. O. (2021a). Genome mining of three plant growth-promoting *Bacillus* species from maize rhizosphere. *Appl. Biochem. Biotechnol.* 193 1–21. 10.1007/s12010-021-03660-3 34529229 PMC8610958

[B34] Olanrewaju O. S. Babalola O. O. (2019a). Bacterial consortium for improved maize (*zea mays* l.) production. *Microorganisms* 7:519. 10.3390/microorganisms7110519 31683950 PMC6920993

[B35] Olanrewaju O. S. Babalola O. O. (2019b). *Streptomyces*: implications and interactions in plant growth promotion. *Appl. Microbiol. Biotechnol.* 103 1179–1188. 10.1007/s00253-018-09577-y 30594952 PMC6394478

[B36] Olanrewaju O. S. Oyatomi O. Babalola O. O. Abberton M. (2021b). Genetic diversity and environmental influence on growth and yield parameters of Bambara groundnut. *Front. Plant Sci.* 12:796352. 10.3389/fpls.2021.796352 34987538 PMC8721115

[B37] Olanrewaju O. S. Oyatomi O. Babalola O. O. Abberton M. (2021c). GGE biplot analysis of genotype x environment interaction and yield stability in Bambara groundnut. *Agronomy* 11:1839. 10.3390/agronomy11091839

[B38] Peiffer J. A. Spor A. Koren O. Jin Z. Tringe S. G. Dangl J. L. (2013). Diversity and heritability of the maize rhizosphere microbiome under field conditions. *Proc. Natl. Acad. Sci. U. S. A.* 110 6548–6553. 10.1073/pnas.1302837110 23576752 PMC3631645

[B39] Philippot L. Raaijmakers J. M. Lemanceau P. Van Der Putten W. H. (2013). Going back to the roots: the microbial ecology of the rhizosphere. *Nat. Rev. Microbiol.* 11 789–799. 10.1038/nrmicro3109 24056930

[B40] Qiao Q. Wang F. Zhang J. Chen Y. Zhang C. Liu G. (2017). The variation in the rhizosphere microbiome of cotton with soil type, genotype and developmental stage. *Sci. Rep.* 7:3940. 10.1038/s41598-017-04213-7 28638057 PMC5479781

[B41] Rolfe S. A. Griffiths J. Ton J. (2019). Crying out for help with root exudates: adaptive mechanisms by which stressed plants assemble health-promoting soil microbiomes. *Curr. Opin. Microbiol.* 49 73–82. 10.1016/j.mib.2019.10.003 31731229

[B42] Sharma A. Verma R. K. (2018). “Root–microbe interactions: understanding and exploitation of microbiome,” in *Root Biology*, 1st Edn, eds Giri B. Prasad R. Varma A. (Cham: Springer), 323–339. 10.1007/s11103-015-0417-8

[B43] Shi S. Nuccio E. Herman D. J. Rijkers R. Estera K. Li J. (2015). Successional trajectories of rhizosphere bacterial communities over consecutive seasons. *mBio* 6:e00746. 10.1128/mBio.00746-15 26242625 PMC4526712

[B44] Stopnisek N. Shade A. (2021). Persistent microbiome members in the common bean rhizosphere: an integrated analysis of space, time, and plant genotype. *ISME J.* 15 1–15. 10.1038/s41396-021-00955-5 33772106 PMC8397763

[B45] Sugiyama A. Ueda Y. Zushi T. Takase H. Yazaki K. (2014). Changes in the bacterial community of soybean rhizospheres during growth in the field. *PLoS One* 9:e100709. 10.1371/journal.pone.0100709 24955843 PMC4067361

[B46] Sun A. Jiao X. Y. Chen Q. Trivedi P. Li Z. Li F. (2021). Fertilization alters protistan consumers and parasites in crop-associated microbiomes. *Environ. Microbiol.* 23 2169–2183. 10.1111/1462-2920.15385 33400366

[B47] Wagner M. R. (2021). Prioritizing host phenotype to understand microbiome heritability in plants. *New Phytol.* 232 502–509. 10.1111/nph.17622 34287929

[B48] Wang M. Eyre A. W. Thon M. R. Oh Y. Dean R. A. (2020). Dynamic changes in the microbiome of rice during shoot and root growth derived from seeds. *Front. Microbiol.* 11:559728. 10.3389/fmicb.2020.559728 33013792 PMC7506108

[B49] Weits D. A. Van Dongen J. T. Licausi F. (2021). Molecular oxygen as a signaling component in plant development. *New Phytol.* 229 24–35. 10.1111/nph.16424 31943217

[B50] Wonglom P. Suwannarach N. Lumyong S. Ito S.-I. Matsui K. Sunpapao A. (2019). Streptomyces angustmyceticus nr8-2 as a potential microorganism for the biological control of leaf spots of *Brassica rapa* subsp. pekinensis caused by *Colletotrichum* sp. and *Curvularia lunata*. *Biol. Control* 138:104046. 10.1016/j.biocontrol.2019.104046

[B51] Xiong C. Singh B. K. He J.-Z. Han Y.-L. Li P.-P. Wan L.-H. (2021a). Plant developmental stage drives the differentiation in ecological role of the maize microbiome. *Microbiome* 9:171. 10.1186/s40168-021-01118-6 34389047 PMC8364065

[B52] Xiong C. Zhu Y. G. Wang J. T. Singh B. Han L. L. Shen J. P. (2021b). Host selection shapes crop microbiome assembly and network complexity. *New Phytol.* 229 1091–1104. 10.1111/nph.16890 32852792

[B53] Xu T. Cao L. Zeng J. Franco C. M. Yang Y. Hu X. (2019). The antifungal action mode of the rice endophyte *Streptomyces hygroscopicus* osish-2 as a potential biocontrol agent against the rice blast pathogen. *Pestic. Biochem. Physiol.* 160 58–69. 10.1016/j.pestbp.2019.06.015 31519258

[B54] Zheng Y. Feng Z. Wang J. Huang X. Lei L. Zhang X. (2021). Wheat-root associated prokaryotic community: interplay between plant selection and location. *Plant Soil* 464 183–197.

