# Supplementary material for: Plant Growth Stage Drives the Temporal and Spatial Dynamics of the Bacterial Microbiome in the Rhizosphere of Vigna subterranea
Source: Front Microbiol. 2022 Feb 17;13:825377. doi: 10.3389/fmicb.2022.825377 (PMC8891599; doi:10.3389/fmicb.2022.825377)
Supplement: Supplementary file 2 [file Presentation_1.PPTX]

## Slide 1
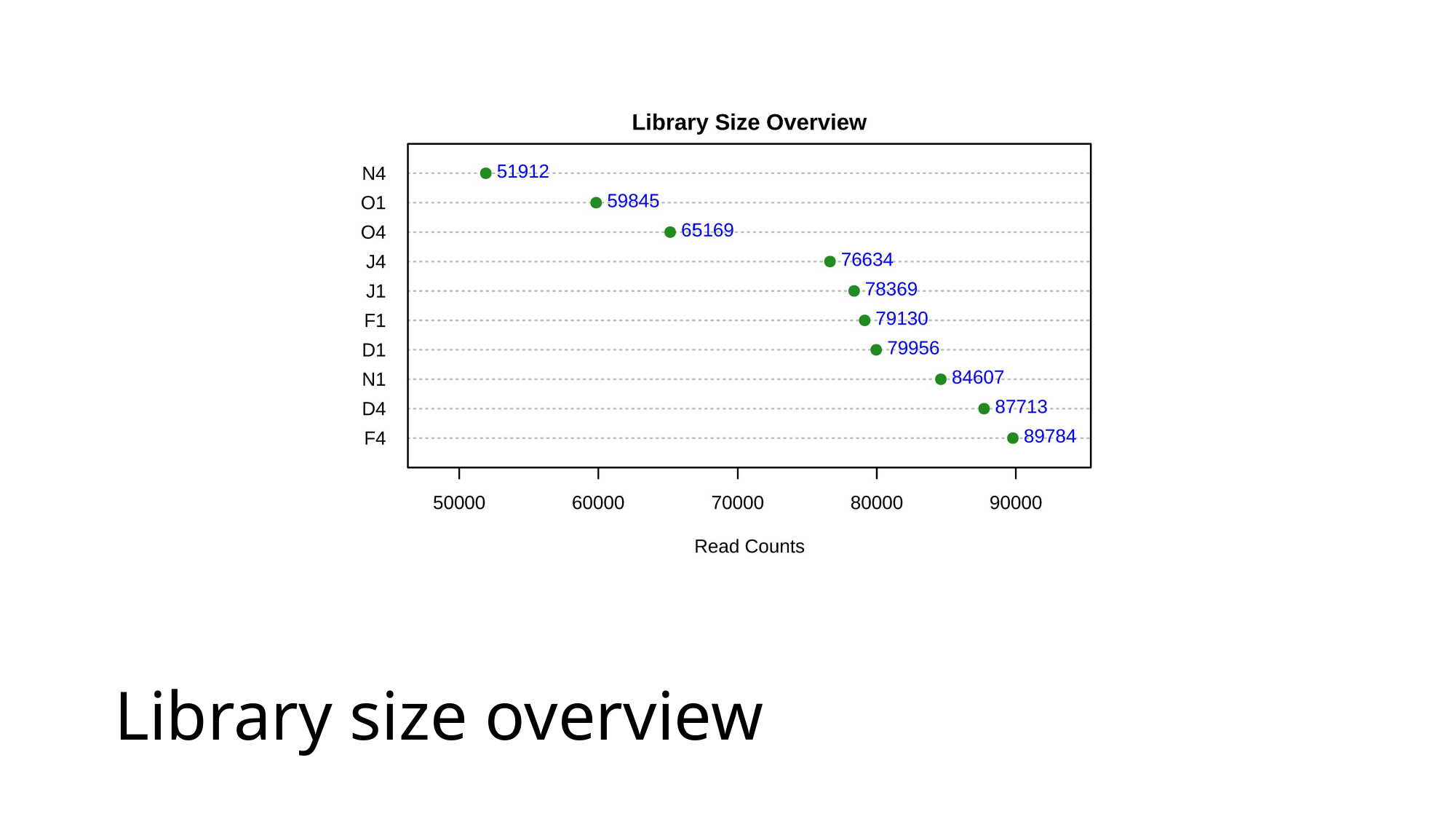

# Library size overview

## Slide 2
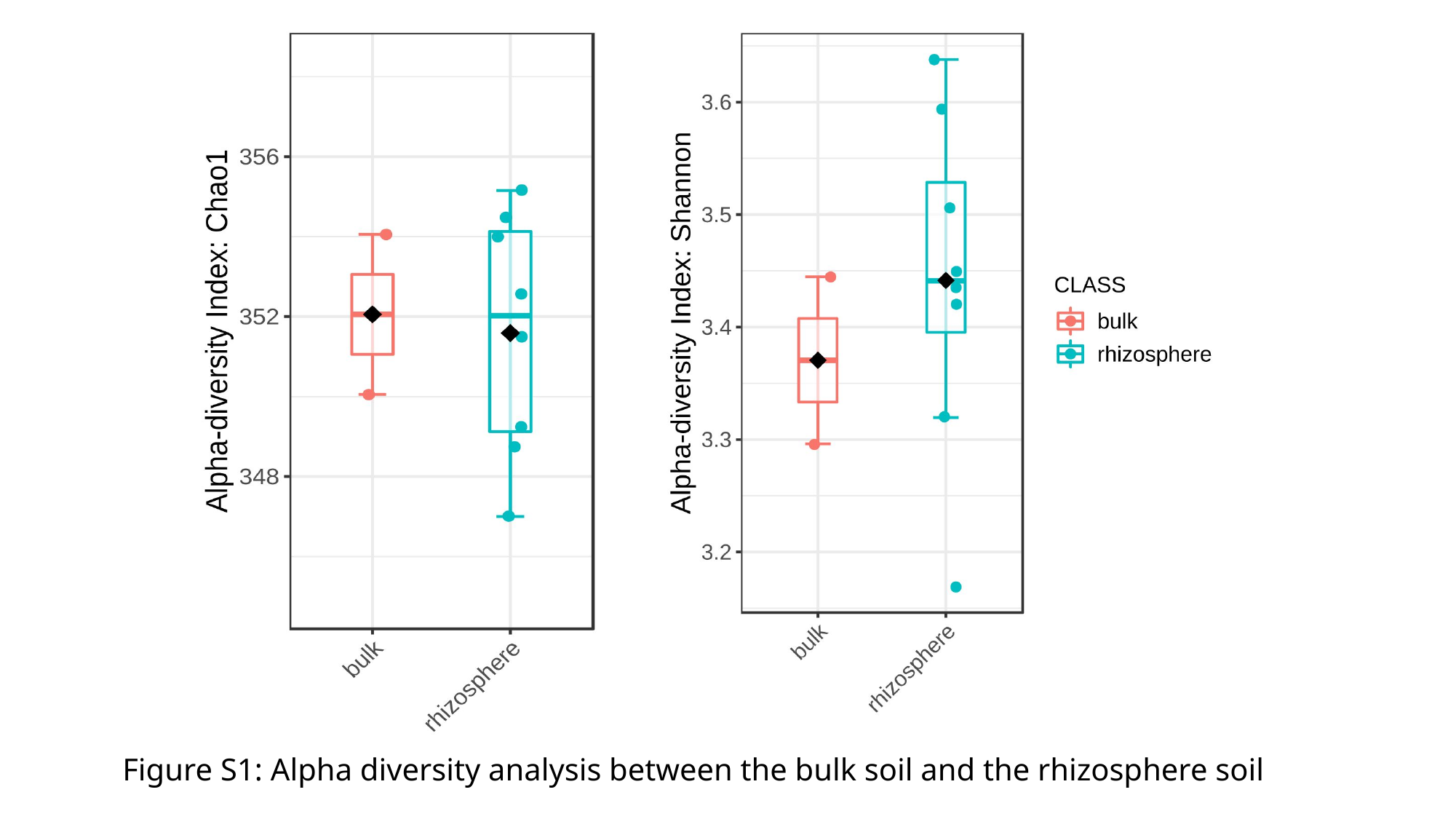

# Figure S1: Alpha diversity analysis between the bulk soil and the rhizosphere soil

## Slide 3
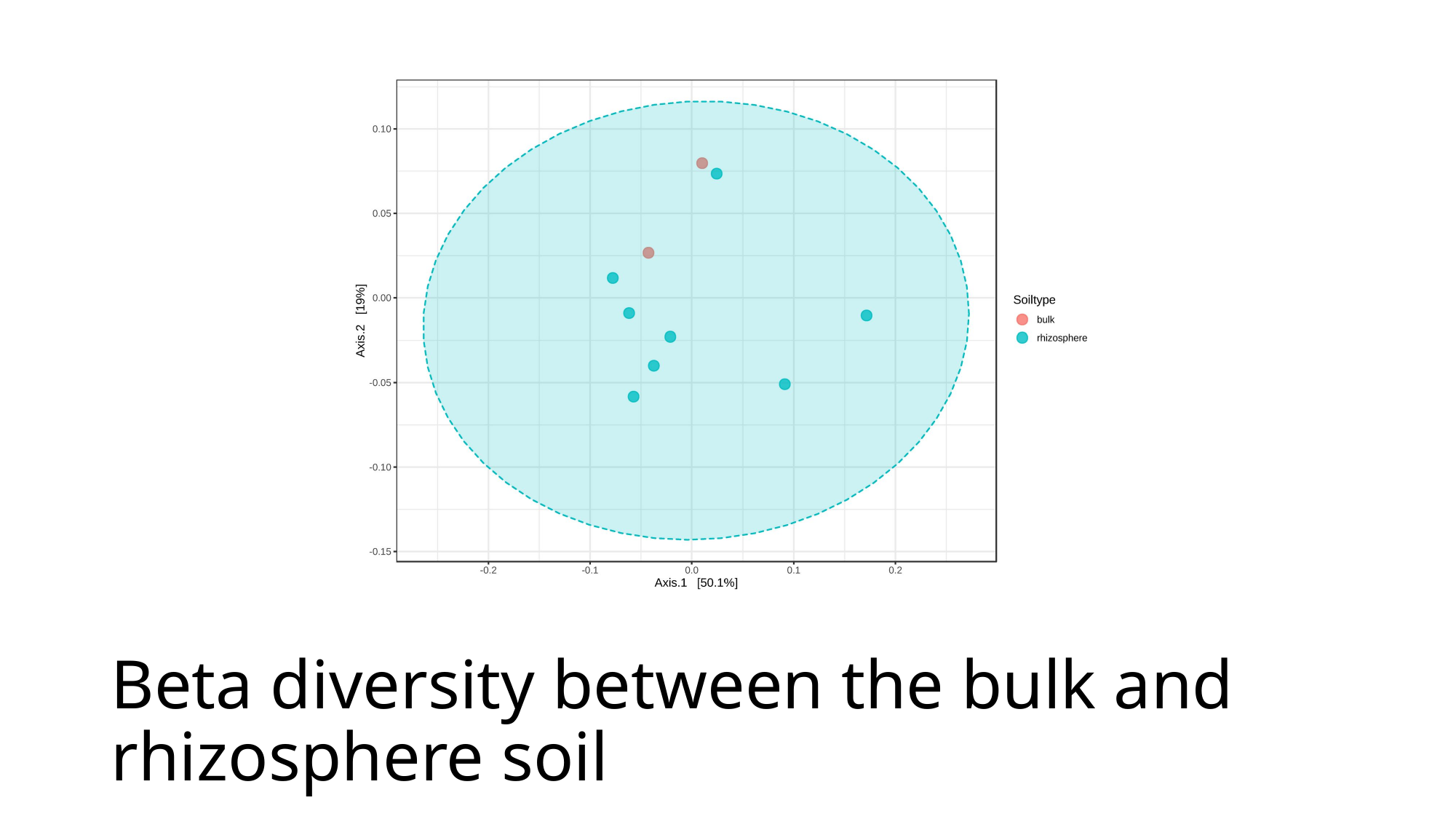

# Beta diversity between the bulk and rhizosphere soil

## Slide 4
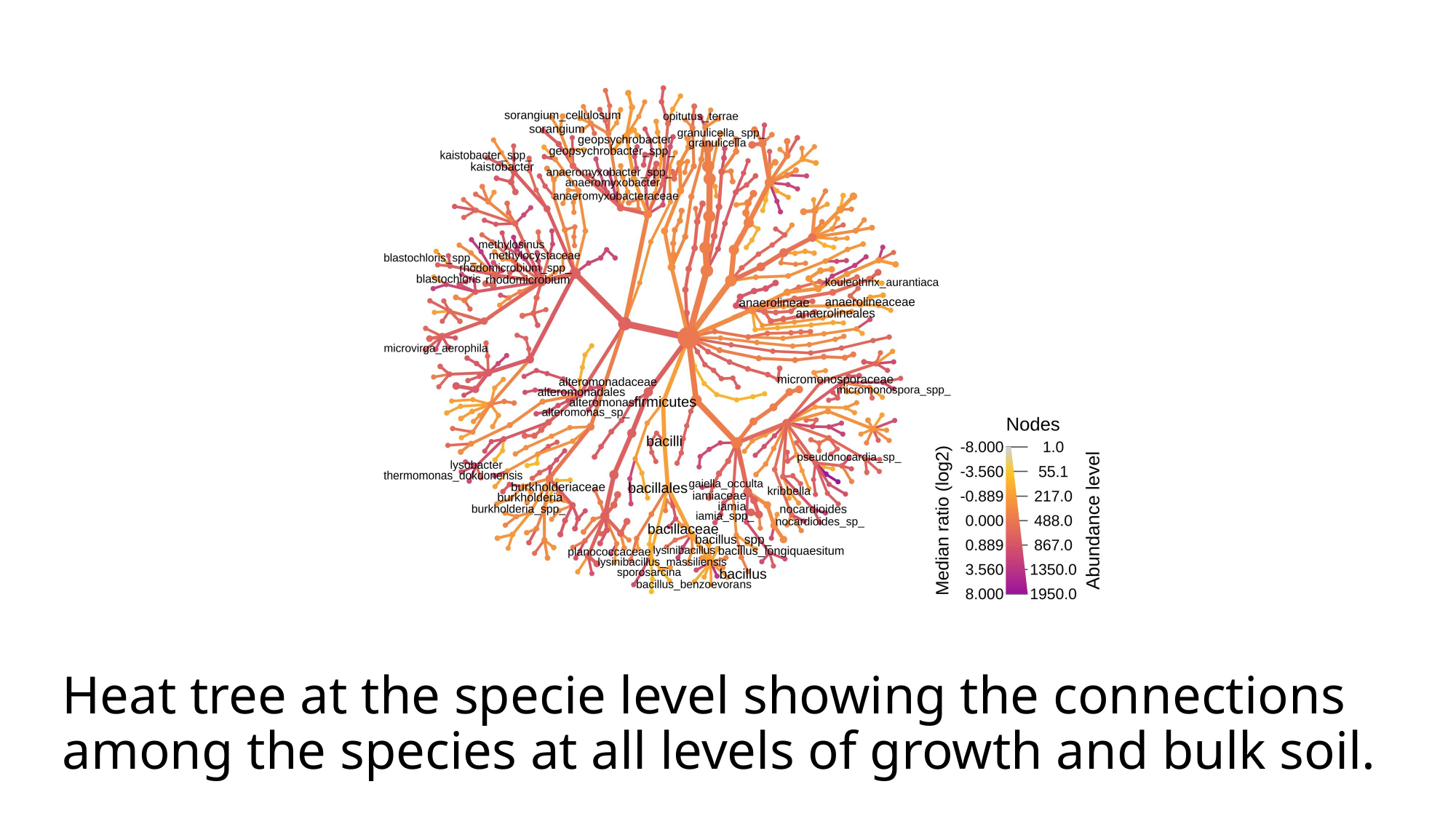

# Heat tree at the specie level showing the connections among the species at all levels of growth and bulk soil.

## Slide 5
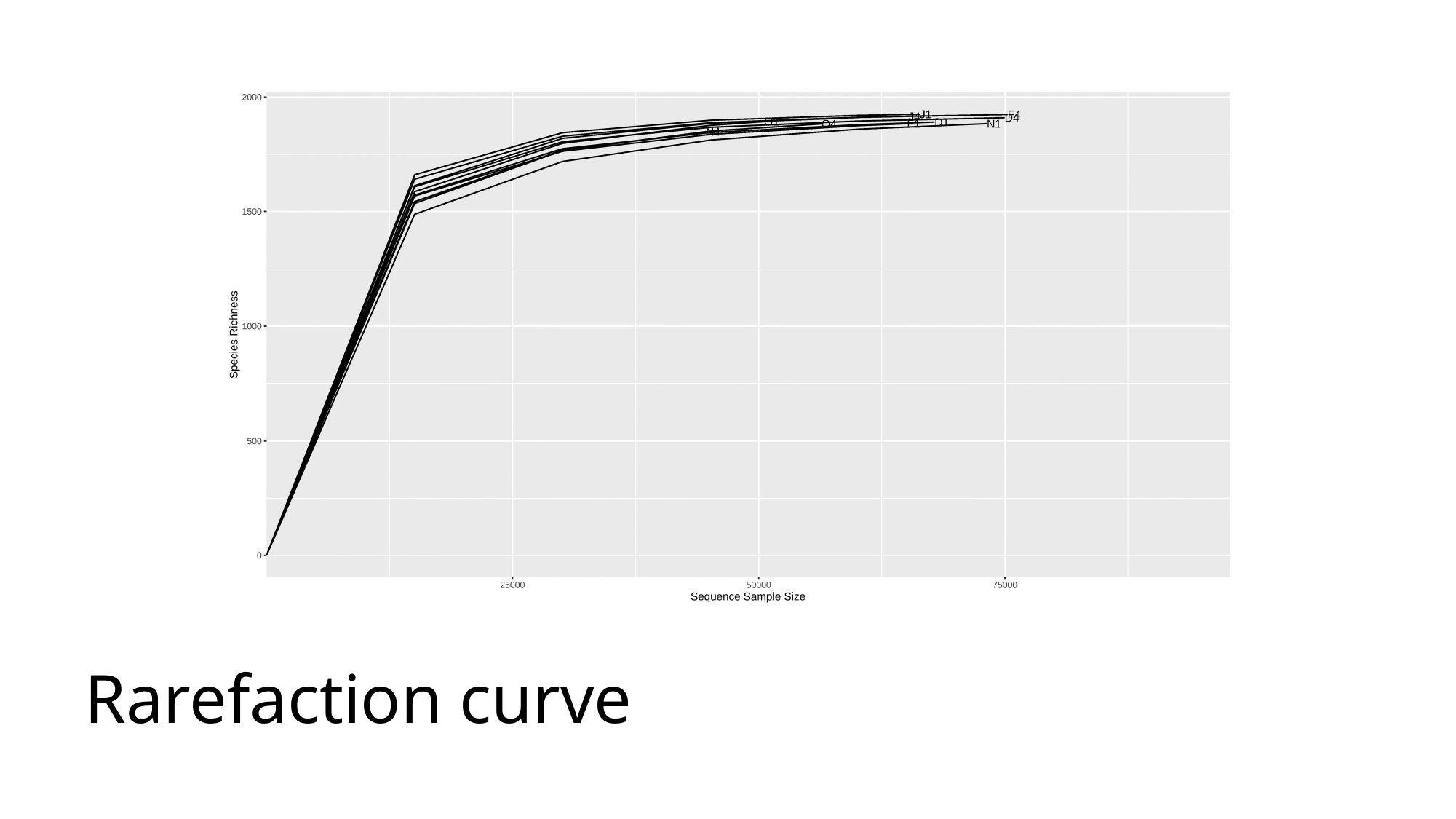

# Rarefaction curve

## Slide 6
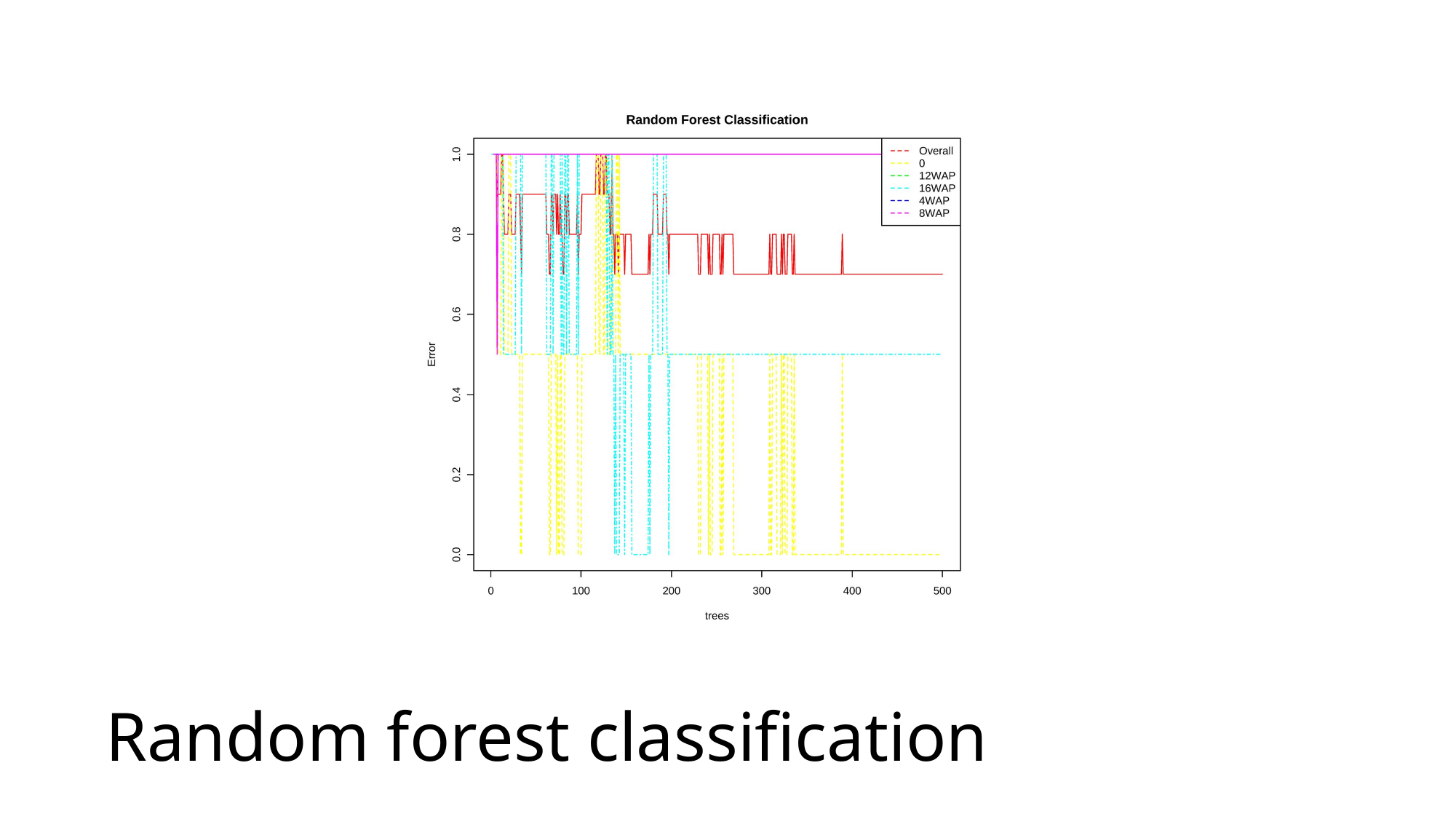

# Random forest classification
